# How UK surgeons experience burnout and the link between burnout and patient care: A qualitative investigation

**DOI:** 10.1177/00369330221122348

**Published:** 2022-09-07

**Authors:** Tmam Al-Ghunaim, Judith Johnson, Chandra Shekhar Biyani, Daryl B O’Connor

**Affiliations:** 1 School of Psychology, 4468 University of Leeds, Leeds, UK; 2 Department of Urology, 4472 St James’s University Hospital, Leeds, UK; 3 Bradford Institute for Health Research, Bradford Royal Infirmary, Bradford, UK; 4 School of Public Health and Community Medicine, University of New South Wales, Sydney, Australia

**Keywords:** Burnout, surgeons, patient care

## Abstract

**Background and Aims:**

Poor well-being affects the performance of all kinds of workers, including surgeons. This study aimed to answer two questions: (1) how does burnout affect surgeons personally, and what is their burnout experience like? (2) How does burnout affect the care that surgeons provide in the United Kingdom (UK)?

**Method:**

This study conducted thematic analysis of semi-structured interviews with 14 surgeons recruited from the UK National Health Service (NHS).

**Result:**

The study found three themes in surgeons’ experiences of burnout: first, burnout is common but frequently not recognised nor understood; second, burnout is a personal crisis; and third, burnout creates vulnerability at work. The study also revealed four themes related to burnout's effect on patient care: first, burnout reduces the quality of surgeon-patient relationships; second, burnout affects patient safety; third, burnout impairs staff relationships; and fourth, burnout makes surgeons less motivated to improve.

**Conclusion:**

Burnout is common but not well recognised in surgeons. Improving understanding and treatment of burnout could have benefits for both surgeons themselves and the care they provide to patients.

## Introduction

Burnout syndrome is characterised as a state of emotional weariness, depersonalisation, and a sense of personal failure that eventually leads to a loss of productivity at work.^1^ According to a recent systematic review and meta-analysis of 89 independent studies, surgeons report higher levels of burnout than their non-surgical peers.^2^ In addition, compared to other medical professionals, surgeons are less likely to seek help.^3^

Concerningly, another recent systematic review and meta-analysis with a total of 27,248 surgeons found burnout was linked to a higher chance of making a surgical or medical error.^4^ The review also investigated professionalism, which can be understood as the behaviours of professionals. While the professionalism outcome factors were too varied for meta-analysis, a narrative synthesis of these revealed a relationship between high burnout and a higher likelihood of loss of temper, malpractice litigation, and a lack of empathy.^4^ Taken together, these findings indicate that burnout in surgeons could have implications for patient care and that addressing surgeon burnout may be one route to delivering higher quality, safer patient care.

There has been a growing literature investigating rates of burnout in surgeons^5–7^ and the factors which are associated with medical errors.^8–10^ However, while the relationship between burnout and patient safety is now established, the mechanisms underlying this relationship are unclear. Understanding this could advance the science of burnout and help identify areas where burnout interventions could usefully be delivered to both reduce surgeon burnout and diminish the risk of poor patient care in response to burnout. Qualitative investigations are particularly useful when seeking to understand complex relationships between variables, but few qualitative studies have been conducted into surgeon burnout, and these have focussed on the drivers behind burnout.^11^ As such, the present study focuses on (1) understanding the experience of burnout from the perspective of surgeons; and (2) exploring the relationship between surgeon burnout and the quality and safety of patient care.

## Methodology

### Research design

This study used a qualitative approach, namely semi-structured face-to-face or telephone interviews, to gain an in-depth understanding of the topic and to acquire first-hand perspectives of surgeons’ experiences. The data used in this publication were part of a larger study that looked into the relationship between burnout and the patient care.^12^ The study followed the SRQR (Standards for Reporting Qualitative Research), which consists of 21 elements.^13^

### Interview creation

A proforma was used to collect data on the participants, including their grade, speciality, number of years as a surgeon, and the type of hospital they worked in. The whole interview schedule (Appendix A) was divided into three sections: (1) burnout definitions and experiences (2) contributors to burnout and coping mechanisms, and (3) how burnout may impair patient care quality and safety. The current research focussed on the data from the first and third sections.

### Data collection

Purposive sampling was used, which is a technique that is frequently used when a diverse sample is required.^14^ Because burnout was expected to differ between specialties, the sample included surgeons from a variety of specialties with varying levels of expertise. The first author spoke at four surgeon gatherings in various specialties working in the UK, discussing the study in general and asking surgeons who wanted to participate in an interview to provide their contact information. The information was also gathered through social media platforms such as Twitter, with doctors from a variety of surgical specialties from throughout the UK being urged to take part in the interview. Five tweets with the hashtags “surgeons,” “UK,” and “NHS” were sent out at different times as part of the Twitter campaign.

This study had 14 participants and yielded a large amount of data to meet the study's objectives. In addition, the number of participants followed the usual guidelines for sample size in terms of data saturation and information power.^15^ To preserve confidentiality, the interview was recorded and then removed from the recording equipment within 72 h. The data was collected between August 8, 2019, and January 15, 2020.

### Ethics

The ethics committee of the School of Psychology at the University of Leeds approved this study (reference number: PSC-674; accepted on 06/05/2019).

### Procedure

Participants who chose to participate were asked when they would like the interview to take place. Their consent was documented over the phone (n = 11 for telephone interviews) or as a signed hardcopy prior to the start of each interview (n = 3 for face-to-face interviews). The interviews lasted 45 min on average (range: 35–90 min). For taking part, each participant received a certificate (Appendix B).

### Data analysis

Braun and Clark's five-step paradigm for thematic analysis was used in this study, which included (1) data familiarisation, (2) initial code development, (3) theme searching, (4) name and definition, and (5) report production.^16^

This type of analysis was chosen because it could yield unexpected insights into the factors that contribute to surgeon burnout and how they cope with it. Two reviewers (TG, a psychology PhD candidate, and JJ, a psychology lecturer, and HCPC-registered Clinical Psychologist) coded two transcripts separately and compared their results to enable triangulation of analysis. TG coded the remaining transcripts. The themes were then double-checked and fine-tuned at researcher meetings, including the whole team (TG, JJ, DOC, SB).

## Results

### Participants

A total of 14 surgeons were interviewed (11 males: 78.57 per cent; 3 females: 21.43 per cent). There were nine consultants and five registrars (Table 1). Nine white participants, three mixed-race participants, and two Asian participants made up the ethnically diverse sample. The participants’ surgical experience ranged from 22.5 to 14.78 years (mean = 22.5; SD = 14.78).

**Table 1.** Demographics of participants.

Name	Gender	Grade	Speciality	Experience as surgeon
Interview 1	Male	Consultant	Neurosurgery	25 years
Interview 2	Male	Consultant	Urology	45 years
Interview 3	Female	Registrar	Urology	5 years
Interview 4	Male	Consultant	Urology	40 years
Interview 5	Female	Registrar	Urology	10 years
Interview 6	Male	Consultant	Urology	19 years
Interview 7	Male	Consultant	Urology	33 years
Interview 8	Female	Registrar	Colorectal Surgery	7 years
Interview 9	Male	Consultant	Otorhinolaryngology	22 years
Interview 10	Male	Consultant	Otorhinolaryngology	44 years
Interview 11	Male	Consultant	Neurosurgery	23 yeas
Interview 12	Male	Registrar	Neurosurgery	4 years
Interview 13	Male	Registrar	Neurosurgery	4 years
Interview 14	Male	Consultant	Plastic surgery	39 years

**Table 2.** Themes, subthemes, example of thematic analysis I: How does burnout effect surgeons personally?

Themes	Subthemes	Example
Theme One: Burnout Is Common but Frequently Not Recognized nor Understood	• Acknowledging that burnout is a spectrum	“I think we all experience it to varying degrees. I experienced burnout in all of my training” (Interview 11).
	• Burnout is common for surgeons	“I think Surgeons are more affected by burnout because of the nature of their work. Other specialties in medicine, there is evidence of burnout, there is a chance of burnout but because of the stress of the job as it is in surgical specialties. The burnout become much more prominent for us and much more often” (Interview 2).
	• Burnout can be subconscious	"Maybe sometimes subconsciously, without realizing that they are burnt out. I think it can be overlooked sometimes” (Interview 11).
	• Wellbeing is the absence of problems	"Wellbeing, in my definition, is the feeling that you are working without stress” (Interview 11).
	• Missing the shades of grey—the lower ends of the burnout spectrum	"I expected my directors to give some advice as to how I can manage the situation, because I didn‘t I didn‘t recognize at that time that I was under a lot of stress. I expected my directors to give some advice as to how I can manage the situation, because I didn‘t recognize at that time that I was under a lot of stress” (Interview 5).
	• Surgeons disregard their burnout	“They either person try to ignore or just think we surgeons doesn‘t happen to us” (Interview 1).
Burnout is a personal crisis	• Social isolation	“Some people will become more reclusive and avoid all social events” (Interview 13).
	• Depression	“I think it can lead to depression.” (Interview 12).
	• Anger	"I did have one thing where I told somebody to stop working on the computer, and come on help me please. I got cross at one moment. That was it. I apologized immediately.” (Interview 10).
Theme Three: When Burnout Creates Vulnerabilities at Work	• Training is a vulnerable time	"I was burnt out to a certain extent, and training to the point that I resigned. I experienced burnout in all of my training,” (Interview 11).
	• The problem of surgeon burnout has increased recently	"I think that especially with, you know, changes in pension law and so on, our careers are being made to be longer and longer than previously they were. And I think that that means you’re going to have to deal with more change towards the end of your career” (Interview4).
	• Surgeons are more comfortable talking about burnout which occurred in the past	"The single most stressful event I’ve had was a complaint to the fitness-to-practice at GMC, by a patient. A lot of years ago. Probably 15 years ago.” (Interview4).

**Table 3.** Themes, subthemes, example of thematic analysis II: How does burnout affect surgeon provided care?

Themes	Subthemes	Example
Theme one: The Effect of Burnout on Surgeon-Patient Relationships and Surgeon-Patient Communication	• Surgeons do not follow up with patients because of burnout	“If I’m stressed about this patient, I want to go and try. I am more worried about it, you know what I mean? I want to see them more. But, I’m not sure that‘s a common and it‘s certainly not a universal reaction. I’ve been aware of something that colleagues do, when things go wrong. They sort of turn their backs on the situation” (Interview 4).
	• Burnout affects patient communication	“Obviously you become very defensive and yourself, whatever you want to do will not be able to do. And. That makes you feel that you are not complete. So it makes you feel a bit shaky. It makes you unsure about what you’re offering your patients and what to ask them to feel.it is effect on your performance, and if the complication which happen that of course you have to go and explain for the patients family, why this happen?, you know may be yes, it might affect the relationship, but it might affect the outcome”(Interview 2).
Theme Two: Patient Safety (Burnout Increases the Risk of Errors)	• Burnout negatively affects decision-making, especially with less experienced surgeons	“When I was very tired, I wasn‘t as quick to make decisions as well as I normally would” (Interview 5).
	• Burnout affects patient harm and medical error	"I felt that I don‘t want to be part of an unsafe practice, basically, and I resigned. Because it becomes unsafe, basically. It‘s not healthy for the surgeon and it becomes unsafe for patients as well” (Interview 11).
	• Note keeping	“when people not interested probably they lazier and not do as much” (Interview 3).
	• Reduced learning from complications	"You’d be less bothered by complications. I’d be less likely to reflect on the situation and think about the next time” (Interview 13).
	• Double checking is a strategy for patient safety from burnout	“Well, I think certainly took extra time. I spent a lot longer with patients trying to explain stuff, than I normally would have done. Just cause it was taking me longer to explain basically. And, then it‘s just a case of checking and double checking, triple checking your notes, decisions, et cetera, that you actually documented the right thing, listed the right person for the right stuff. It‘s a case of double checking things” (Interview 5).
Theme Three: Burnout Affects Staff Relationships (Burnout Negatively Affects Colleague Interactions or Teamwork)	• Surgeon burnout affects their relationships with family and colleagues	“ you’re more likely to get angry at your colleagues or patience is shorter” (Interview 12).
	• Surgeon burnout contributes to the use of ego defence mechanisms	“Everyday life brings something that you will not always know how to cope with it, therefore one needs to be prepared and have various mechanisms to cope. Makers of defence mechanisms to fight for that sort of thing” (Interview 7).
	• Teamwork buffers the impact of burnout on safety	“As I said, about having colleagues to be able to discuss cases with. If you discuss with colleagues that are understanding, then they can give you advice and point out areas where you might have to arrange a particular test or a way of doing things differently. It‘s about having good communication with your work colleagues, I think” (Interview 9).
Theme four: Burnout Makes Surgeons Less Motivated to Improve	• Less open to learn	“So, when it‘s not perfect, usually I’ll think about, maybe I’ll change that and make small tweaks rather than big changes. So, I think it‘s all about thinking how you could be bothered about even the small complications, so that you can perfect it. So, if you are burned out, you don‘t think of that. It‘s about getting home quicker” (Interview 13).

## Thematic analysis - part one

The first section of the analysis focused on the responses to the question, “How does burnout affect surgeons personally?” Findings were grouped under three main themes: (1) Burnout is common but frequently not recognised nor understood; (2) Burnout is a personal crisis; (3) When burnout creates vulnerability at work.

### Theme one: Burnout is common but frequently not recognised nor understood

This theme captured that although surgeons commonly recognise burnout in others, paradoxically, they struggle to recognise it in themselves until it is severe.

Some surgeons regarded burnout as a continuum or spectrum. Along the trip, there are various but distinct stops, each of which builds on the previous one:

“I think we all experience it to varying degrees. I experienced burnout in all of my training” (Interview 11).

“Yes, sometimes I was exhausted and tired, but I did not think that I lost focus lost interest"(Interview 7).

Working as a surgeon can be demanding, with early starts and late finishes, as well as increased duties. This led some surgeons to describe emotional exhaustion and burnout as a common affliction:

“I think Surgeons are more affected by burnout because of the nature of their work. Other specialties in medicine, there is evidence of burnout, there is a chance of burnout but because of the stress of the job as it is in surgical specialties. The burnout become much more prominent for us and much more often. (Interview 2)”

Although burnout was described as common, some surgeons were not fully aware of whether they were burned out or not. This was possibly because of a lack of awareness of the burnout concept, or probably because some surgeons had a lack of insight into themselves and were not aware of their feelings:

“Maybe sometimes subconsciously, without realising that they are burnt out. I think it can be overlooked sometimes. (Interview 11)”

"No, I think surgeons are not very good at analysing themselves. So, I think they probably carry on and probably don‘t recognise things. Or, maybe just a human thing. But, I think surgeons are particularly bad at, probably just continuing despite. Because we’re driven by the work and the need to do things. And, the fact we’ve got this job that we have to keep doing and can‘t just stop. (Interview 10)"

Some surgeons failed to understand burnout and thought that wellbeing was a life without problems or issues:

"Wellbeing, in my definition, is the feeling that you are working without stress. (Interview 11)”

Some surgeons felt that unless it was having a serious impact on their work, they were not experiencing burnout. Believing that burnout meant extreme suffering, some surgeons missed the shades of grey—the lower ends of the burnout spectrum.

"I expected my directors to give some advice as to how I can manage the situation, because I didn‘t I didn‘t recognise at that time that I was under a lot of stress” (Interview 5).

Sometimes, surgeons said that when they had identified that they had burnout, they ignored it and disregarded the issue of burnout.

“They either person try to ignore or just think we surgeons don‘t happen to us" (Interview 1).

### Theme two: Burnout is a personal crisis

The experience of burnout among surgeons affected them socially, which included them isolating themselves, expressing feelings of depression, and easily getting angry. As a result of burnout, their social relationships were also affected. Some surgeons mentioned that when they faced burnout, they tended to isolate themselves, lose their interest in social contact, and recoil into themselves:

“Some people will become more reclusive and avoid all social events.” (Interview 13)

Some surgeons described the effect of burnout as having depression:

“I think it can lead to depression.” (Interview 12)

Some surgeons described that burnout led them to easily lose their temper with their patients. Many surgeons also stated that they expressed their anger through interactions with their colleagues/co-workers.

“I did have one thing where I told somebody to stop working on the computer, and come on help me please. I got cross at one moment. That was it. I apologised immediately. (Interview 10)”

### Theme three: Burnout creates vulnerability at work

Some surgeons argued that when they have burnout, it has a negative effect on their work. They revealed that the problem of burnout has increased in the years and months prior to the time of the interview (which was before the Covid pandemic). In addition, surgeons seemed to be more comfortable talking about past experiences of burnout than their present feelings relating to burnout.

Some surgeons mentioned that they suffered more from burnout during their training, which they considered a very stressful time.

"I was burnt out to a certain extent, and training to the point that I resigned. I experienced burnout in all of my training” (Interview 11).

Some surgeons stated that the problem of burnout had increased over time, linked with policy changes:

"I think that especially with, you know, changes in pension law and so on, our careers are being made to be longer and longer than previously they were. And I think that that means you’re going to have to deal with more change towards the end of your career” (Interview 4).

Many surgeons were more open and felt more comfortable talking about previous burnout experiences rather than new ones. As a result, surgeons rarely disclosed current concerns, possibly due to the vulnerability such a disclosure may have generated:

"The single most stressful event I’ve had was a complaint to the fitness-to-practice at GMC, by a patient. A lot of years ago. Probably 15 years ago. (Interview4)"

Another surgeon also mentioned this: "Like I said, this was the main event in my career, where I experienced burnout in a true major form, back in 2000” (Interview 11).

## Thematic analysis - part two

The second section of the analysis focused on the responses to the question, how does burnout affect surgeons providing care? Findings fell under four main themes: (1) the effect of burnout on surgeon-patient relationships and surgeon-patient communication, (2) patient safety (burnout increases the risk of errors), (3) burnout affects staff relationships (burnout negatively affects colleague interactions or teamwork), and (4) burnout makes surgeons less motivated to improve.

### Theme one: Burnout reduces the quality of surgeon-patient relationships

Many surgeons described how burnout affected surgeon-patient relationships. For example, they described that when surgeons are suffering from burnout, they tend to avoid contact with patients in order to avert confrontations and protect themselves from the stress of these types of interactions:

“If I’m stressed about this patient, I want to go and try. I am more worried about it, you know what I mean? I want to see them more. But, I’m not sure that‘s a common and it‘s certainly not a universal reaction. I’ve been aware of something that colleagues do, when things go wrong. They sort of turn their backs on the situation.” (Interview 4)

“Obviously you become very defensive and yourself, whatever you want to do will not be able to do. And. That makes you feel that you are not complete. So it makes you feel a bit shaky.” (Interview 2)

However, some surgeons reported that this strategy could backfire, with some patients becoming increasingly angry if they did not see them:

“That can help people tend to be more short or angry or cut corners. Yes, definitely. (Interview 3)”

To manage the stress of these difficult interactions when they were feeling burnt out, some surgeons described making efforts to reduce this issue and help their relationships with patients by trying to relax before seeing them:

"I just make sure that before I interact with the patient in any way that I am in a calm situation. And if I think I’m not, then I will take a couple of minutes out, have a coffee or something as a coping mechanism” (Interview14).

### Theme two: Patient safety (burnout increases the risk of errors)

This theme captured the association surgeons identified between burnout and an increased risk of making wrong decisions, medical errors, note-keeping errors or being unaware of or missing complications. Some surgeons described how burnout could lead to indecisiveness or making incorrect decisions. They found this a concerning impact of burnout, because, as surgeons, they recognised that their decisions could have direct and significant effects on patients. Making the wrong decisions could lead to a patient safety incident:

“When I was very tired, I wasn‘t as quick to make decisions as well as I normally would [be].” (Interview 5)

When burnout led to patient safety incidents, surgeons described feeling emotionally distressed and experiencing a sense that they had lost control:

"I felt that I don‘t want to be part of an unsafe practice, basically, and I resigned. Because it becomes unsafe, basically. It‘s not healthy for the surgeon and it becomes unsafe for patients as well” (Interview 11).

“I realised that I wasn‘t recognising that this was happening to me. And surgeons have a way of trying to just deal with things on our own. We tried to solve our own problems. And essentially, I think there comes a point where all of these things are happen and you have a straw that breaks the camel‘s back, so to speak. So you sort of you end up having an incident in theatre (Interview 14)”

Inadequate note-keeping was also reported to be another consequence of surgeon burnout with the potential to negatively impact patient care:

“When people not interested probably they lazier and not do as much” (Interview 3).

However, while some surgeons experienced distress in response to incidents which occurred when they were burnt out, others reported caring less, feeling less concerned about patients’ wellbeing and reduced learning from complications:

"You’d be less bothered by complications. I’d be less likely to reflect on the situation and think about the next time. (Interview 13)”

Recognising the link between burnout and less safe patient care, some surgeons used a double-checking strategy to try to protect their patients:

“Well, I think certainly took extra time. I spent a lot longer with patients trying to explain stuff, than I normally would have done. Just because it was taking me longer to explain basically. And, then it‘s just a case of checking and double checking, triple checking your notes, decisions, et cetera, that you actually documented the right thing, listed the right person for the right stuff. It‘s a case of double checking things” (Interview 5).

### Theme three: Burnout affects staff relationships (burnout negatively affects colleague interactions or teamwork)

This theme captured the association between burnout and teamwork. Some surgeons described how burnout impaired their communication with their colleagues and caused them to lose their temper more frequently with colleagues:

“You’re more likely to get angry at your colleagues or patience is shorter.” (Interview 12)

When surgeons felt burnt out, some described using ego-defence mechanisms to become more aggressive in order to protect themselves:

“Everyday life brings something that you will not always know how to cope with it, therefore one needs to be prepared and have various mechanisms to cope. Makers of defence mechanisms to fight for that sort of thing.” (Interview 7).

However, other surgeons described drawing on their teams when they felt burnt out, in order to mitigate the risk that their burnout could lead patients to receive poorer quality or less safe patient care. For example, one surgeon reported discussing cases with fellow team members rather than making decisions alone, recognising that their decision-making capacity may be reduced and they may need external support as a result of experiencing burnout:

"As I said, about having colleagues to be able to discuss cases with. If you discuss with colleagues that are understanding, then they can give you advice and point out areas where you might have to arrange a particular test or a way of doing things differently. It‘s about having good communication with your work colleagues, I think.” (Interview 9)

### Theme four: Burnout makes surgeons less motivated to improve

This theme described how burnout made some surgeons less motivated to improve and less open to learning; as a result, when they had burnout, some surgeons described themselves as having less development potential and were less motivated to improve:

"So, when it‘s not perfect, usually I’ll think about, maybe I’ll change that and make small tweaks rather than big changes. So, I think it‘s all about thinking how you could be bothered about even the small complications, so that you can perfect it. So, if you are burned out, you don‘t think of that. It‘s about getting home quicker. (Interview 13)”

Whereas others became less open to learning:

"I think in terms of interacting and learning from other colleagues” (Interview 13).

## Discussion

This study aimed to answer two specific questions. Firstly, how does burnout affect surgeons personally, and what is the experience like for them? Secondly, how does burnout affect the care that surgeons provide in the UK? Three main themes were identified as being important in surgeons’ experience of burnout: (1) burnout is common, but it is frequently unrecognised or misunderstood; (2) burnout is a personal crisis; (3) burnout creates vulnerability at work. In addition, four areas were revealed relating to the effect that burnout has on the care that surgeons provide: (1) surgeon-patient relationships, (2) patient safety, (3) staff relationships (burnout negatively affects colleague interactions or teamwork) and (4) burnout makes surgeons less motivated to improve.

These findings support studies that indicate that burnout is a common problem in surgeons.^5–7^ Also, our data support a similar finding reported by Gerada and Jones, which found that surgeons are less likely to seek help than other medical professionals.^3^ The current study highlights that some surgeons find it difficult to recognise burnout in themselves and when they claimed that after recognising that they were burnt out, they neglected the problem. This may be considered as a way of escaping their negative feelings by ignoring whether there is a problem or not, to protect their ego. Similarly, these findings are consistent with larger studies that reported burnout has been linked to several negative personal outcomes of surgeons, including a link to greater levels of depression^17–20^ and anxiety.^18^

These findings also support the studies which described that trainee surgeons have a significantly higher risk for burnout than attending surgeons (a physician who is board-certified or board-qualified in surgery) in a variety of specialties.^2,5,21^ In addition, surgical trainees have been found to be at a higher risk of burnout than consultants or attending surgeons.^5,22^ A systematic review carried out by Galaiya, Kinross and Arulampalam showed that burnout levels are higher in people who are less experienced.^23^ Therefore, there is clearly a need for more and higher-quality studies investigating burnout treatments in surgeons and surgical trainees.^24^

The second question this study attempted to answer was: how does burnout affect the care that surgeons provide? These findings support studies that have found that burnout has a negative impact on patient satisfaction and surgical professionalism.^6^ Similarly, a systematic review reported that surgeon burnout affects surgeons’ professionalism, including a higher risk of loss of temper and malpractice suits and lower empathy.^4^ Nine separate studies and the present study suggest significant links between surgeon burnout and patient safety.^9,17,25–30^

The present study also extends existing knowledge by revealing that burnout can have a negative effect on collegiate interactions and teamwork, similar to that of Copeland, who also described that burnout significantly affected surgeon communication skills.^31^ In addition, our results add to existing knowledge by identifying that burnout may make surgeons less motivated to improve. This is in line with findings reported by Yavari, Ismaeli, and Rezaie, who highlighted a significant relationship between overall burnout and motivation.^32^

### Implications

The current study also has implications for burnout interventions. Most interventions are aimed to prevent rather than treat mental illness, and most research is related to physicians in general rather than specific specialisations or career stages.^32^ The method in which interventions are implemented appears to be critically important. The mentality and mindset of pragmatic surgery professionals may be more compatible with an intervention approach that focuses on “what can be done” to deal with stress and burnout at work.^24^ The findings from our study suggest interventions should be designed to be low-stigma as we found that surgeons were reluctant to talk about current burnout. Resilience-building training may therefore be less stigmatising than interventions promoted as “burnout support” interventions and more acceptable to surgeons. Similarly, our study indicates that surgeons may not recognise the earlier stages of burnout. Perhaps interventions should focus on awareness-raising to help surgeons identify when they are experiencing work-related stress.

### Strengths and limitations

It is important to acknowledge the strengths and limitations of the current study. A strength of this study is that the qualitative methodology used provides a deep insight into how surgeons experience burnout and its effect on them. Another strength is that surgeons from different surgical specialty and of diverse grades were approached. Even though many quantitative studies have been carried out relating to surgeon burnout and medical errors,^8–10^ few contribute towards a better and richer understanding of this problem.

However, this study does have some limitations. One of these is that there was a gender imbalance within the sample, with only three women were interviewed, as opposed to 11 men. According to Sutherland et al., female surgeons are five times more likely to report burnout when compared with males.^33^ Future research ought to try to ensure that equal numbers of male and female surgeons are recruited. A second limitation is that the study relied wholly on one-to-one interviews, rather than including focus groups. This approach was chosen to enable flexibility in the times when interviews were conducted and to reduce the burden of the study for surgeons. It was also chosen to ensure privacy and confidentiality for participants. However, a previous study on general practitioners used focus groups and found this to be a rich data collection method.^34^ Future qualitative research in surgeons may benefit from also including focus groups, alongside interviews.

In conclusion, this study concentrated on key themes relating to surgeons’ burnout experiences: their understanding of burnout, how they cope with it, and how it affects their relationships with others. Four specific areas linked to surgeon burnout were also identified: how it affects relationships with patients, interactions and teamwork, patient safety, and surgeon motivation. Our findings suggest that hospital managers need to take action to reduce burnout to ensure that patients receive the best care possible and to reduce medical errors. More preventive intervention programmes are needed to help surgeons understand and recognise the problem of burnout and improve their wellbeing to help improve patient safety.
